# Structural and Thermal Characterization of Milled Wood Lignin from Bamboo (*Phyllostachys pubescens*) Grown in Korea

**DOI:** 10.3390/molecules29010183

**Published:** 2023-12-28

**Authors:** Ji-Sun Mun, Sung-Phil Mun

**Affiliations:** 1 Department of Carbon Materials and Fiber Engineering, Jeonbuk National University, Jeonju 54896, Republic of Korea; jismun20@jbnu.ac.kr; 2 Department of Wood Science and Technology, Jeonbuk National University, Jeonju 54896, Republic of Korea

**Keywords:** bamboo, *Phyllostachys pubescens*, milled wood lignin, structural analysis, thermal analysis

## Abstract

The structural and thermal characterization of milled wood lignin (MWL) prepared from bamboo (*Phyllostachys pubescens*) grown in Korea was investigated, and the results were compared with bamboo MWLs from other studies. The C_9_ formula of the bamboo MWL was C_9_H_7.76_O_3.23_N_0.02_ (OCH_3_)_1.41_. The Mw and Mn of MWL were 13,000 and 4400 Da, respectively, which resulted in a polydispersity index (PDI) of 3.0. The PDI of the prepared MWL was higher than other bamboo MWLs (1.3–2.2), suggesting a broader molecular weight distribution. The structural features of MWL were elucidated using FT-IR spectroscopy and NMR techniques (^1^H, ^13^C, HSQC, ^31^P NMR), which indicate that MWL is of the HGS-type lignin. The major lignin linkages (β-O-4, β-β, β-5) were not different from other bamboo MWLs. The syringyl/guaiacyl ratio, determined from ^1^H NMR, was calculated as 0.89. ^31^P NMR revealed variations in hydroxyl content, with a higher aliphatic hydroxyl content in MWL compared to other bamboo MWLs. Thermal properties were investigated through TGA, DSC, and pyrolysis-GC/MS spectrometry (Py-GC/MS). The DTG_max_ of MWL under inert conditions was 287 °C, and the T_g_ of MWL was 159 °C. Py-GC/MS at 675 °C revealed a syringyl, guaiacyl, *p*-hydroxyphenyl composition of 17:37:47.

## 1. Introduction

According to the Food and Agriculture Organization (FAO), the annual rate of deforestation was estimated at 10 million ha between 2015 and 2020. Over the past three decades, an estimated 420 million ha of forest have been lost since 1990 [1]. This rampant forest destruction contributes to approximately 20% of global carbon emissions [2]. Moreover, the excessive use of wood has faced strong criticism due to the continuous depletion of forest resources worldwide [3]. As the world’s wood resources decline, there is a growing need for lignocellulosic biomass to replace them. Among these lignocellulosic biomass, bamboo is an emerging biomass that can replace wood because of its short production cycle and high amount of biomass per unit area. In addition, the remarkable regeneration capability and minimal maintenance requirements make bamboo an environmentally friendly alternative [3,4,5]. The Food and Agriculture Organization (FAO) reports that bamboo is widely grown in tropical and subtropical climatic zones, including in East, Southeast, and South Asia [6]. In historical China, bamboo found diverse applications, such as arrowheads, baskets, writing scrolls, pens, paper, boats, shoes, and construction materials [7]. In modern-day China, bamboo utilization spans various applications, including bamboo shoots, ceiling/flooring interiors, scaffolding, timber, furniture, and crafts [3,8]. According to China’s 9th National Forest Inventory, China’s bamboo forest area gradually increased from 2014 to 2018, with a total bamboo forest area of 6.73 million ha [6]. In this light, China’s research on bamboo remained active over these years. However, in Korea, bamboo consumption is declining, leading to the desolation of domestic bamboo forests [9]; hence, there has not been much research on bamboo in Korea.

Bamboo can be processed into a variety of products, including particleboards, plywood, laminated bamboo, bamboo composites, and bamboo fiber [10,11,12]. Truly, the utilization of bamboo is limited due to its hollow structure, and certain bamboo stems may not be affected by certain types of chemicals due to their waxy skin [13]. These impermeable properties may limit chemical treatment to improve mechanical properties for potential applications [3]. In addition to these problems, numerous obstacles still impede the efficient utilization of bamboo resources. Therefore, overcoming these hurdles requires further research efforts. 

On the other hand, bamboo can be easily delignified compared to other woody biomass [14,15]. In other words, it offers the advantage of producing pulps, as well as lignin, more readily compared to other conventional methods. Additionally, bamboo lignin contains a substantial amount of *p*-hydroxyphenyl units, a feature nearly absent in softwood and hardwood lignins [16]. The high reactivity of this unit, with vacant positions at 3 and 5, is expected to significantly contribute to future lignin utilization.

In this study, the structural and thermal characteristics of lignin were investigated as a fundamental study for the future utilization of bamboo grown in Korea. In addition, the results obtained were compared with previously reported bamboo lignin.

## 2. Results and Discussion

### 2.1. Chemical Composition of Bamboo

Analysis of ash, extracts, carbohydrates, and lignin content was performed to determine the chemical composition of bamboo, and the results are shown in Table 1. The alkaline extract yield was approximately 30%, surpassing that of cold-water, hot-water, and organic solvent extracts. Li et al. [17] investigated changes in the maturation of bamboo (*Phyllostachys pubescens*). The ash, Klason lignin, holocellulose, and α-cellulose content were close to the reported values of 3-year-old bamboo culms.

### 2.2. Chemical and Structural Characterization of Bamboo MWL

#### 2.2.1. Elemental Composition of MWL

The C, H, O, N, S, and OCH_3_ contents of MWL are shown in Table 2. The methoxyl content was estimated from the integration ratio of protons originating from aromatic rings and methoxyl groups in the ^1^H NMR, following the method outlined by Abreu [18]. The C, H, and O contents of the MWL prepared in this study were similar to MWL-A from *Phyllostachys acuta*. On the other hand, the C content of MWL was lower than that of MWL-X from an unknown bamboo species. The methoxyl content of MWL was found to be higher than the other two bamboo MWLs. This result may be due to the presence of fewer *p*-hydroxyphenyl (H) units, which have no methoxy groups, in the two bamboo lignins, as shown in Table 2 for comparison. In Table 3, the C_9_ molecular formula and formula weight of MWL and other bamboo MWLs are listed. The formula weight of MWL was approximately 214 Da, higher than that of the other two bamboo MWLs.

#### 2.2.2. Molecular Weight (MW) Distribution, Average MW, and Polydispersity

**Figure 1:**
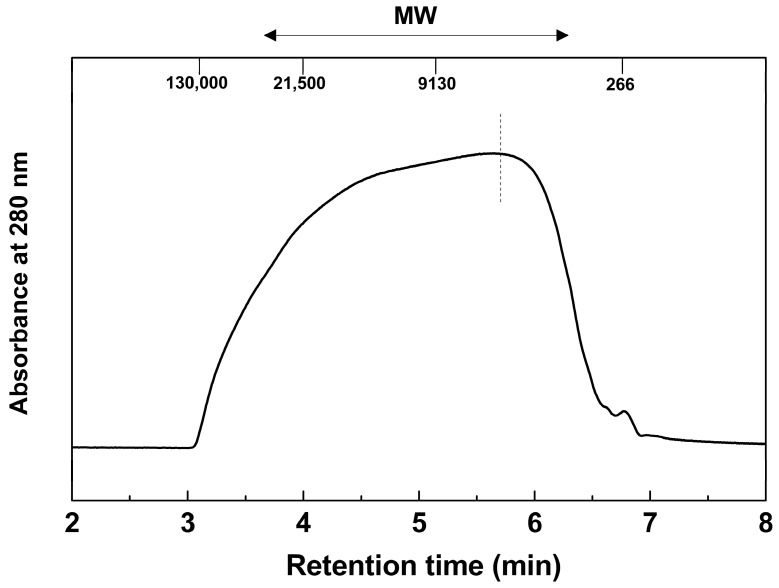
MW distribution of Ac-MWL.

The weight-average ($\bar{M}$w) and number-average ($\bar{M}$n) MW, along with the polydispersity index (PDI), of acetylated MWL (Ac-MWL), were determined using gel permeation chromatography (GPC). The MW distribution of Ac-MWL is illustrated in Figure 1, and the corresponding $\bar{M}$w, $\bar{M}$n, and PDI values are presented in Table 4. For comparison, data on previously reported bamboo MWLs are provided in Table 4. The $\bar{M}$w and $\bar{M}$n of Ac-MWL were approximately 13,000 Da and 4400 Da, respectively. The $\bar{M}$w of Ac-MWL prepared in this study was higher than that of all other bamboo MWLs. In addition, the PDI of Ac-MWL was also higher than that of all other bamboo MWLs, indicating a broader distribution of MW. The Mw, Mn, and PDI differed even from the same bamboo species. This result is likely attributed to the different climatic zones wherein the bamboo was grown, since the Korean-grown bamboo is from temperate regions and the Chinese-grown bamboo is from subtropical regions. The Korean-grown bamboo was believed to have thicker and more rigid culms to withstand colder temperatures and harsher climatic conditions. This robustness was thought to contribute to the greater strength and density of Korean-grown bamboo, potentially resulting in a higher Mw.

#### 2.2.3. FT-IR Spectroscopy

**Figure 2:**
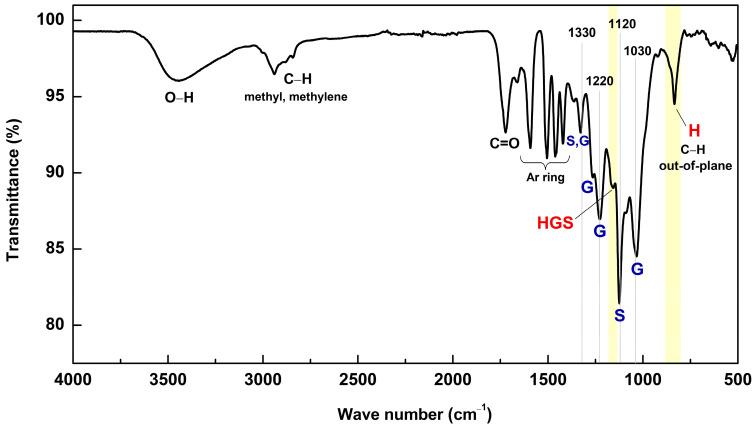
FT-IR (ATR) spectrum of MWL.

FT-IR spectroscopy was conducted for the determination of functional groups in MWL. In Figure 2, the FT-IR spectrum of MWL is presented. The bands have been assigned according to the work of Faix [24], and the assignments are detailed in Table 5. The observed spectral features of MWL aligned with those of HGS-type lignins found in other bamboo MWLs [19,21,24]. Notably, the band at 834 cm^−1^, corresponding to C–H out-of-plane vibrations in H units, along with a characteristic shoulder at 1160 cm^−1^—typical for HGS type—was observed. In addition, the C=O stretching related to unconjugated ketone, carbonyl, and ester groups was assigned at 1718 cm^−1^, aromatic skeleton vibrations at 1594, 1503, and 1419 cm^−1^, syringyl (S)-related bands at 1130 and 1123 cm^−1^, and guaiacyl (G)-related bands at 1330, 1266, 1222, and 1033 cm^−1^.

#### 2.2.4. ^1^H NMR Analysis

**Figure 3:**
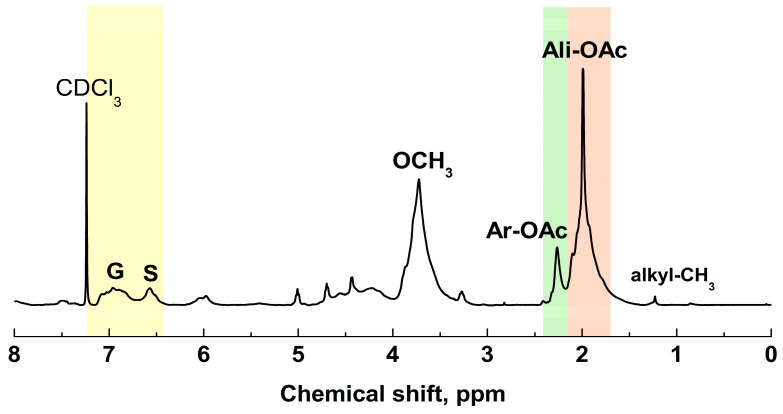
^1^H NMR spectrum of Ac-MWL.

The ^1^H NMR spectrum of Ac-MWL is presented in Figure 3. The aromatic region (7.20–6.25 ppm) revealed the presence of G and S phenylpropane (C_9_) units. The aliphatic content was higher than the aromatic content.

Signal assignments in the ^1^H NMR spectrum of Ac-MWL are listed in Table 6, based on literature data [25,26]. To estimate the distribution of protons per C_9_ structural unit in Ac-MWL, integration ratios and their C_9_ molecular formulas were used [27]. The methoxyl content in the C_9_ molecular formula for MWL was 1.41, multiplied by 3 to yield 4.23—the total number of protons in the methoxyl groups. Integration values for other structural components were normalized to the methoxyl protons in a single C_9_ unit. However, certain quantitative conclusions cannot be drawn due to overlapping signals, carbohydrate inclusions, and uncertainties in range assignments.

The arylglycerol β-O-4 aryl ether linkage (6.25–5.75 ppm, 4.90–4.30 ppm) is the main intermonomeric linkage found in native lignin [25]. The H_α_ and H_β_ contents from Ac-β-O-4 structures were highest among the linkages. The aromatic protons per C_9_ unit for Ac-MWL were determined to be 0.93 for S units and 1.05 for G units. The S/G molar ratio of MWL based on ^1^H NMR was determined to be 0.89.

To ascertain the number of aliphatic and phenolic hydroxyl groups per C_9_ unit, corresponding acetyl group signals (2.50–1.60 ppm) were examined. The Oac/OCH_3_ mole ratio was calculated as (0.76 + 4.38)/4.23, resulting in 1.22. Consequently, the total Oac/C_9_ ratio was determined as (1.41 OCH_3_) × (1.22 Oac/1 OCH_3_), yielding 1.72. The number of aliphatic Oac/OCH_3_ was calculated as (1.41 OCH_3_/C_9_) × (4.38 Oac/4.23 OCH_3_), resulting in 1.46, while the number of phenolic Oac/OCH_3_ was calculated as (1.41) × (0.76/4.23), resulting in 0.25. Therefore, the estimated number of aliphatic and phenolic hydroxyl groups per 100 C_9_ units of MWL was 146 and 25, respectively.

#### 2.2.5. ^13^C NMR Analysis

**Figure 4:**
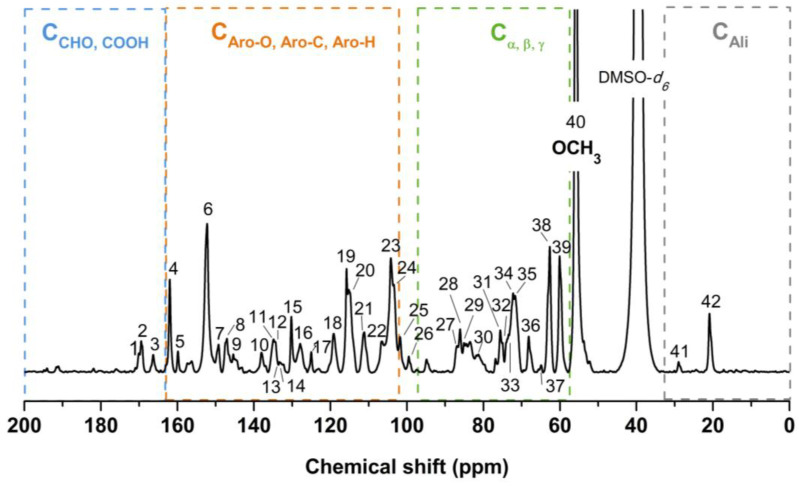
^13^C NMR spectrum of MWL.

The structural features and linkages within MWL were further elucidated through ^13^C NMR analysis. In Figure 4, the ^13^C NMR spectrum of MWL is presented, and Table 7 provides the chemical shifts along with their assignments based on previously reported works [16,21,28,29]. The NMR spectrum was divided into four regions: C=O, aromatic, side chain, and aliphatic.

The signals at positions **1** and **2** correspond to carbonyl groups in MWL. Signals at positions **25**, **26**, **31**, **32**, and **33** were attributed to residual carbohydrates, which likely originated from impurities such as traces of hemicelluloses associated with some MWL substructures. The intense signal at position **40** is indicative of OCH_3_ in both S and G units.

In the aromatic region (166 to 103 ppm) of the spectrum, several peaks were assigned to S, G, and H units. For S units: C-3/C-5 etherified (**6**), C-5 nonetherified (**8**), C-4 etherified (**10**), C-1 etherified and C-4 nonetherified (**11**), C-1 nonetherified (**14**), C-2/C-6 with α-carbonyl (**22**), C-2/C-6 (**23**, **24**). For G units: C-4 etherified and C-3 etherified G with α-carbonyl (**7**), C-3 (**8**), C-4 nonetherified (**9**), C-1 etherified (**12**), C-1 nonetherified (**13**), C-6 (**18**), C-5 (**20**), C-2 (**21**). For H units: C-4 (**4**), C-2/C-6 (**16**), and C-3/C-5 (**20**). Additionally, six peaks were assigned to C-9 (**3**), C-4 (**5**), C-2/C-6 (**15**), C-1 (**17**), C-3/C-5 (**19**), and C-8 (**20**) in *p*-coumaric ester (*p*-CE). The NMR analysis indicated that a considerable amount of *p*-coumaric acid (*p*-CA) is etherified at the γ-position [21,30,31,32,33]. The ^13^C NMR results confirmed that MWL is an HGS-type lignin, consistent with the FT-IR results.

The etherified S/nonetherified S unit ratio was estimated based on the peak height ratio at 152.2/147.1 ppm, while the etherified G/nonetherified G ratio was derived from resonance ratios at 149.4/145.5 ppm [34]. The values of etherified S/nonetherified S (4.4) and etherified G/nonetherified G (1.5) suggested a greater involvement of S units in ether linkages with other lignin units compared to G structures. This finding agrees with previously reported results [21,34].

In the side chain region (87 to 57 ppm) of the spectrum, several peaks were assigned to β-O-4, β-β resinol, β-1, and β-5. For β-O-4: C-α (**33**, **34**), C-β (**28**, **30**), C-γ (**39**), C-γ in β-O-4 with α-carbonyl (**38**); for β-β: C-α (**29**), C-γ (**35**), C-4 (**23**); for β-1: C-α (**32**); and for β-5: C-α (**27**), C-γ (**37**, **38**). No notable differences were observed in the ^13^C NMR results of MWL compared to other bamboo MWLs.

Since MWL is a macromolecule, some overlapping signals were observed in the ^13^C NMR spectrum. Therefore, 2D HSQC NMR analysis was performed to enhance spectral resolutions in intercoupling bonds and linkages within lignin substructures.

#### 2.2.6. 2D HSQC NMR Analysis

**Figure 5:**
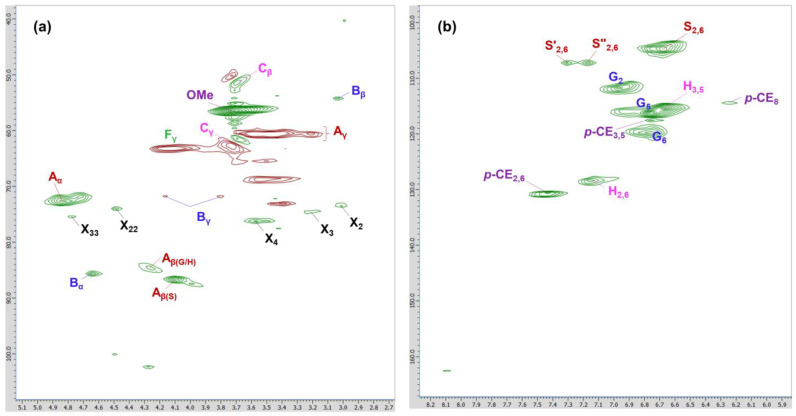
(**a**) Side chain and (**b**) aromatic region of the HSQC spectra of MWL.

**Figure 6:**
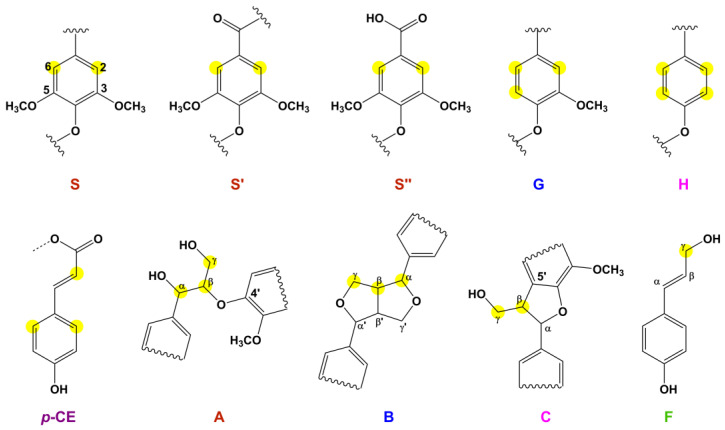
Substructures present in MWL from 2D HSQC NMR.

Figure 5 depicts the side chain (120–70/5.0–3.0 ppm) and aromatic (160–100/8.0–6.0 ppm) regions of MWL in the HSQC spectrum. The aliphatic region was excluded from the discussion, as notable information was not provided. Cross-signals and their assignments, derived from previously reported works by Wen et al. [21,35,36], are shown in Table 8. The substructures present in MWL, along with their corresponding notations, are presented in Figure 6.

In the side chain region (Figure 5a), β-O-4 (A), β-β resinol (B), and β-5 phenylcoumarans (C) moieties were detected. For β-O-4 moieties, C_α_–H_α_ (A_α_) at 72.8/4.85 and C_γ_–H_γ_ (A_γ_) at 60.2/3.38–3.89 were observed. C_β_–H_β_ in β-O-4 substructures linked to a G/H unit (A_β(G/H)_) and S unit (A_β(S)_) were at 84.6/4.26 and 86.6/4.10 ppm, respectively. The signals at 85.8/4.64 and 54.1/3.04 were assigned to C_α_–H_α_ (B_α_) and C_β_–H_β_ (B_β_), respectively, whereas 71.8/4.16 and 72.1/3.80 were assigned to C_γ_–H_γ_ (B_γ_) in β-β moieties. For β-5 moieties, C_β_–H_β_ (C_β_) at 51.5/3.70 and C_γ_–H_γ_ (C_γ_) at 62.8/3.73 were observed. The signals of methoxyl and the β-O-4 moiety were prominent in this region. Additionally, the signal at 63.3/4.11 ppm was assigned to C_γ_–H_γ_ in *p*-hydroxycinnamyl alcohol end-groups (F_γ_). Carbohydrate-associated signals were also found, including signals from β-_D_-xylopyranoside moieties (X_2_, X_3_, X_4_) and 2-O- and 3-O-acetyl-β-_D_-xylopyranoside moieties (X_22_, X_33_), as reported by Kim and Ralph [37] and Wen et al. [35,36].

In the aromatic region (Figure 5b), signals from S, G, and H moieties were highly visible. S moieties (S_2,6_, S′_2,6′_, and S″_2,6_) were in the range of 104.8–107.2/7.30–6.69 ppm. G moieties were situated at 111.9/6.97, 115.4/6.68, and 129.8/6.80 ppm for C_2_–H_2_, C_5_–H_5_, and C_6_–H_6_, respectively. H moieties were observed at 113.8/6.67 ppm for C_3,5_–H_3,5_ and 128.3/7.17 ppm for C_2,6_–H_2,6_. Additionally, three cross signals were assigned to C_3,5_–H_3,5_ (116.1/6.78 ppm), C_8_–H_8_ (116.2/6.26 ppm), and C_2,6_–H_2,6_ (130.6/7.48 ppm) in *p*-CE. The HSQC NMR results confirmed MWL as an HGS-type lignin, consistent with the FT-IR and ^13^C NMR results. Signals related to spirodienone, ferulate, and cinnamaldehyde end-groups were observed in bamboo MWL from *P. pubescens* grown in China [21], but not in bamboo MWL grown in Korea.

#### 2.2.7. ^31^P NMR Analysis

**Figure 7:**
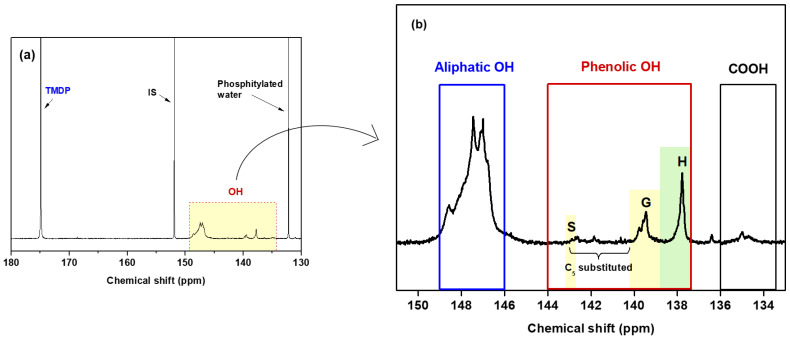
(**a**) ^31^P NMR spectrum and (**b**) enlarged spectrum of MWL.

The hydroxyl and carboxyl content of MWL were determined using the ^31^P NMR method based on Argyropoulos et al. [38], which allows the quantification of different types of hydroxyl groups, including aliphatic and phenolic hydroxyl groups, as well as G, S, H, and C_5_ condensed phenolic hydroxyl groups. In Figure 7a, the full ^31^P NMR spectrum of phosphitylated MWL is presented, and Figure 7b shows the enlarged hydroxyl group region of interest (150–134 ppm). A sharp peak at 174 ppm was attributed to the excess amount of unreacted TMDP, indicating the complete derivatization of all hydroxyl groups in MWL.

The aliphatic and phenolic (C_5_-substituted + S, G, and H) hydroxyl, and carboxyl contents of MWL, calculated from the ^31^P NMR result, are listed in Table 9. The hydroxyl content was compared to other bamboo MWLs based on published data [23,39]. For MWL, the total hydroxyl content was found to be 8.19 mmol/g MWL. The hydroxyl group of the H units was similar in all bamboo MWLs. The aliphatic and total hydroxyl contents in MWL were notably higher, while the C_5_-substituted+S content was lower compared to MWL-Y and MWL-N. The carboxyl content of MWL was determined to be 0.23 mmol/g MWL, close to MWL-N and slightly lower than MWL-Y.

### 2.3. Thermal Characterization of Bamboo MWL

#### 2.3.1. TGA

**Figure 8:**
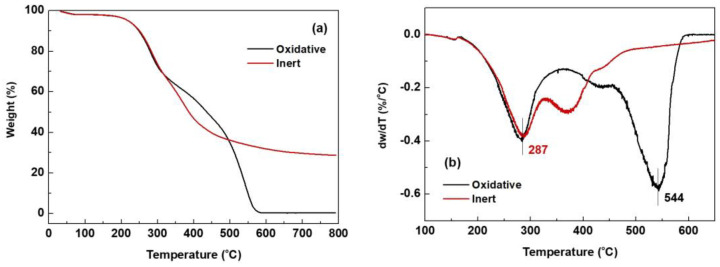
(**a**) TG and (**b**) DTG curves of MWL under oxidative and inert conditions.

TGA is widely employed for examining the thermal behavior and thermal and thermo-oxidative stability of lignin. Figure 8a,b show the thermogravimetric (TG) and derivative TG (DTG) curves of MWL, respectively, under oxidative and inert conditions. In the TG and DTG curves, two crucial temperatures are observed. The onset temperature marks the point at which decomposition begins, indicating the initiation of gas release during thermal oxidation or pyrolysis experiments. The other temperature, DTG_max_, represents the point at which maximum thermal degradation occurs and is considered a parameter for determining the thermal stability of lignins [40].

The decomposition process of the lignin sample can be divided into several stages. In the initial stage before 120 °C, the weight loss is attributed to the evaporation of moisture remaining in the lignin samples [41] and low MW volatiles. Under oxidative conditions at 120 °C, a weight loss of 1.9% in MWL was noted (Table 10). The onset temperature was 234 °C and DTG_max_ was 544 °C. The weight losses at 400 °C and 500 °C were approximately 43% and 65%, respectively. There were no weight changes beyond 590 °C, and the ash content obtained at 800 °C was 0.3%.

Under inert conditions at 120 °C, a weight loss of 1.8% in MWL was observed (Table 10). The onset temperature of MWL was found to be 235 °C, nearly the same as that of oxidative condition (234 °C). In the 200–400 °C region of the DTG curve, two major bands were observed at 287 °C and 370 °C (Figure 8b). The weight loss in this region is attributed to the cleavage of interunit linkages in lignin, releasing monomeric phenols into the vapor phase [42]. Specifically, the peak at 287 °C signifies the degradation of aliphatic side chains, particularly the scission of β-O-4 ether linkages, while the peak at 370 °C indicates the degradation of methoxyl groups [43], based on information regarding gases released during pyrolysis [44]. Between 400 and 600 °C, the weight loss is primarily attributed to the decomposition or condensation of the aromatic ring [42,45]. The weight losses at 400 °C and 500 °C were approximately 11% and 36%, respectively. MWL underwent continuous carbonization at temperatures ranging from 600 to 800 °C, with a residual content of 28.7% at 800 °C.

In Table 10, the DTG_max_ and residual content of other bamboo MWLs are presented in comparison to MWL. The DTG_max_ of MWL was lower (287 °C) than that of other bamboo MWLs (360 and 367 °C). This difference may be attributed to higher S unit content. The residue content of MWL was higher than MWL-Y and lower than MWL-N.

Furthermore, under inert conditions, the DTG_max_ (287 °C) was lower than that obtained under oxidative conditions (544 °C). This can be attributed to the difficulty involved in degrading oxidized condensed aromatic moieties [43].

#### 2.3.2. DSC

**Figure 9:**
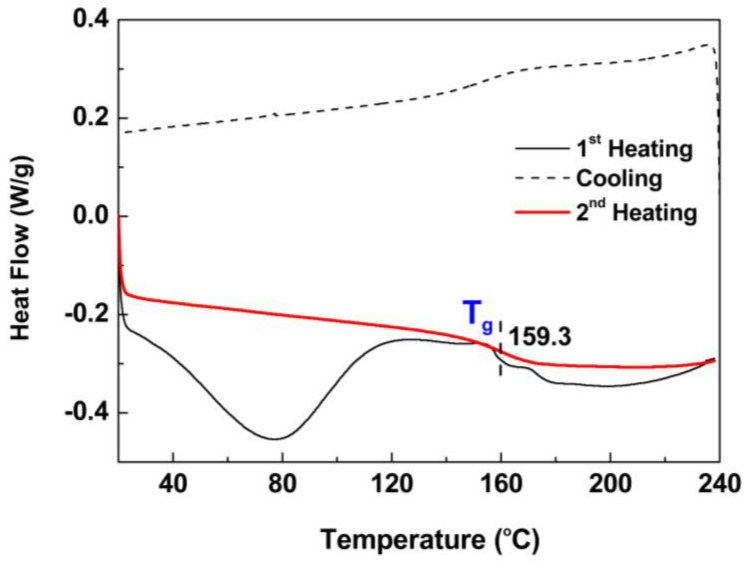
DSC profile of MWL.

The thermal behavior of MWL, expressed as heat flow with respect to temperature, was determined over the range of 20–240 °C. In Figure 9, the 1st heating, cooling, and 2nd heating cycles of MWL are illustrated. During the 1st heating cycle, the thermal history of MWL, i.e., moisture absorbed during storage conditions, residual solvents, and drying methods, was removed. Upon reaching 240 °C, a temperature exceeding its melting transition, MWL was allowed to cool at 20 °C. An exothermic peak observed during the cooling cycle indicated the solidification of the lignin melt. The 2nd heating cycle reveals the true thermal behavior of the sample and is known to provide a reliable estimate of the glass transition temperature (T_g_). T_g_ is an important transition temperature, at which amorphous polymers shift from a glassy to a rubbery state. In the 2nd heating cycle, the T_g_ of MWL was determined to be 159 °C.

#### 2.3.3. Pyrolysis GC/MS (Py-GC/MS)

**Figure 10:**
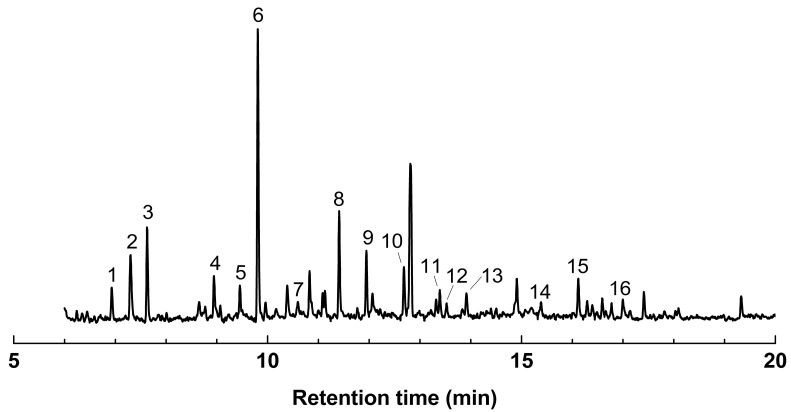
Pyrogram of MWL at 675 °C.

Py-GC/MS was conducted at 675 °C to analyze the composition of MWL. The choice of pyrolysis temperature was based on TGA, where 675 °C was selected due to its minimal observed weight change. Compound identification was achieved by comparing mass spectra with published data from NIST and Wiley libraries [46,47], along with bamboo Py-GC/MS data [20,22,48]. The pyrogram of MWL is shown in Figure 10, and the resulting pyrolysis products with their relative compositions are listed in Table 11. Sixteen monolignol compounds were identified during pyrolysis at 675 °C, encompassing typical H-, G-, and S-related pyrolysis products. The pyrolysis products of MWL were categorized into three groups: H lignin derivatives (peaks 1, 2, 4, 6), G lignin derivatives (peaks 3, 5, 8, 10, 11, 12, 13), and S lignin derivatives (peaks 7, 9, 14, 15, 16). The major pyrolysis products released were 4-vinylphenol (6), 4-vinylguaiacol (8), guaiacol (3), syringol (9), and 4-methylphenol (2). These five major pyrolysis products constituted 68% of the total relative composition of MWL. Among them, 4-vinylphenol was the most abundant, accounting for approximately 30% of the total relative composition, consistent with previously reported findings from Saiz-Jimenez and De Leeuw [48] and Li et al. [20]. The S:G:H composition of MWL was determined to be 16:37:47. The calculated S/G ratio for MWL was 0.43, aligning with a previously published result (0.4) from Bai et al. [22]. The S/G ratio derived from Py-GC/MS differed from the S/G ratios obtained through ^1^H NMR (0.89). However, both methods concurred that the S content was lower than the G content.

## 3. Materials and Methods

### 3.1. Materials

The bamboo powder, prepared from a 2–3-year-old bamboo (*P. pubescens*) culm, was supplied by Songjuk Industry, located in Hamyang, Gyeongnam, Republic of Korea. The bamboo powder was air dried at room temperature for a week, 40 mesh passed powders were used. 

The reagents, sodium hydroxide (NaOH, 93%), sodium chlorite (NaClO_2_, 78%), ethanol (HPLC grade), benzene (EP), acetic anhydride (EP), and tetrahydrofuran (THF, 99.9%) were purchased from Duksan Pure Chemical (Seoul, Republic of Korea). Anhydrous ethyl ether (EP) and acetic acid (EP) were purchased from Samchun Chemical (Seoul, Republic of Korea). 1,2-dichloroethane (GR) and sulfuric acid (H_2_SO_4_, 93%), were purchased from Duksan Pharmaceutical (Sangju-si, Republic of Korea) and Daejung Chemicals & Metals (Siheung, Republic of Korea), respectively. 1,4-Dioxane (HPLC grade) was purchased from Wako Chemical (Tokyo, Japan), anhydrous pyridine (GR) from Kanto Chemical (Tokyo, Japan), toluene (HPLC grade) from Fisher-Scientific Korea (Seoul, Republic of Korea), chloroform-*d* (CDCl_3_) and dimethyl sulfoxide-*d*_6_ (DMSO-*d*_6_) from Eurisotop (Saint-aubin-des-bois, France), *N*-hydroxy-5-norbornene-2,3-dicarboximide (NHND, 97%) from AlfaAesar (Heysham, UK), chromium (III) acetylacetonate (97%) from AlfaAesar (Ward Hill, MA, USA), and 2-chloro-4,4,5,5-tetramethyl-1,3,2-dioxaphospholane (TMDP, 95%) from Sigma-Aldrich (St. Louis, MO, USA). All reagents and solvents were used without further purification. Molecular sieves (pore diameter 4Å, 1.6 mm pellet) were purchased from Yakuri Pure Chemicals (Tokyo, Japan).

### 3.2. Chemical Composition of Bamboo

The ash, extracts, and lignin were measured following TAPPI test methods [49,50,51,52,53,54]. Acid-soluble lignin was determined in accordance with the TAPPI standard method UM 250 [54], using an absorption coefficient of 110 L/g·cm. Holocellulose content was determined using the Wise method [55], and α-cellulose was determined using the TAPPI test method T203 om-83 [56]. Hemicellulose content was calculated by subtracting the α-cellulose value from the holocellulose value.

### 3.3. Preparation of MWL

The thoroughly dried, extractive-free bamboo powder was used for the preparation of MWL. Six grams of bamboo powder were placed in a 500-mL stainless-steel jar and filled with toluene. The jar, containing the sample, was then mounted on a vibratory ball mill and treated for 100 h. After milling, the MWL was isolated and purified according to the Björkman method [57].

### 3.4. Elemental Analysis

The MWL was vacuum dried under P_2_O_5_ at ambient temperature for 24 h prior to elemental analysis. C, H, O, N, and S analyses were performed using an Elemental Analyzer (IT/Flash 2000, Thermo Fisher Scientific, Waltham, MA, USA) at the Center for University-wide Research Facility, Jeonbuk National University (CURF, JBNU).

### 3.5. Acetylation of MWL

For the acetylation, 50 mg of MWL was dissolved in 1 mL of pyridine and 1 mL of acetic anhydride. The reactions of quenching, filtering, washing, and drying were carried out in the same manner as described by Mun et al. [58]. The acetylated MWL was designated as Ac-MWL.

### 3.6. Determination of MW

The average MW of MWL was determined by gel permeation chromatography (GPC). One milligram of Ac-MWL was dissolved in 1 mL of THF in a 10-mL conical beaker. The beaker was sonicated for 5 s and then filtered through a 0.45 μm PTFE syringe filter (Chemco Scientific, Seoul, Republic of Korea). The filtrate was transferred into a 2-mL vial and diluted 2 times with THF. The GPC (Waters, Milford, MA, USA) was conducted at CURF under the conditions shown in Table 12.

### 3.7. FT-IR Spectroscopy

FT-IR analysis was conducted utilizing a diamond attenuated total reflectance (ATR) accessory on an FT-IR spectrophotometer (Frontier, Perkin Elmer, Shelton, CT, USA), equipped with a deuterated triglycine sulfate (DTGS) detector. The spectrum was acquired in the wavelength range of 4000–500 cm⁻¹ with a resolution of 4 cm⁻¹_._ The analysis was performed at the CURF, JBNU. 

### 3.8. ^1^H NMR Analysis

Ten milligrams of the Ac-MWL sample was dissolved in 0.4 mL of CDCl_3_ in a 10-mL conical beaker. The beaker was sonicated for 1–2 min to dissolve the sample. The mixture was filtered through a fine glass wool suspended inside a Pasteur pipette, which was directly connected to a clean NMR tube. The conical beaker was rinsed with additional 0.3 mL of CDCl_3_ and the contents were transferred as described in a previous filtration method. The measurement was conducted using the NMR spectrometer (500 MHz FT-NMR, JNM-ECZ500R, JEOL, Tokyo, Japan) at the CURF, JBNU.

### 3.9. ^13^C and 2D HSQC NMR Analysis

A 120 mg MWL was placed into a 5-mL vial and vacuum dried under P_2_O_5_ at ambient temperature for 24 h before sample preparation. The moisture-free MWL was dissolved at 0.75 mL DMSO-*d*_6_ at 50 °C. The filtration was carried out in the same manner as for ^1^H NMR samples mentioned above. The ^13^C and HSQC NMR analyses were conducted using an NMR spectrometer (600 MHz, JEOL, Tokyo, Japan) at the CURF, JBNU.

### 3.10. ^31^P NMR Analysis

The hydroxyl and carboxyl group content of MWL was determined through ^31^P NMR analysis following the procedure outlined by Argyropoulos et al. [38]. The sample was prepared using pyridine/CDCl_3_ (1.6:1 *v*/*v*) solvent with an internal standard NHND, a relaxation agent (chromium (III) acetylacetonate), and a phosphitylating agent (TMDP). Throughout the process, maintaining a moisture-free condition was crucial. The ^31^P NMR analysis was conducted using an NMR spectrometer (600 MHz, JEOL, Tokyo, Japan) at the CURF, JBNU. The spectrum was obtained using an inverse-gated decoupling pulse sequence, a 10 s relaxation delay, and 64 scans.

### 3.11. TGA

A 4–8 mg MWL was placed in a standard aluminum pan and secured in a thermogravimetric analyzer (Q600 SDT, TA Instruments). The sample was heated from 20 to 800 °C at 10 °C/min under nitrogen and oxidative conditions. TGA was performed at the CURF, JBNU.

### 3.12. DSC

A 2–6 mg MWL was loaded in a standard aluminum pan, and the heat flow was measured by a differential scanning calorimeter (DSC Q20, TA Instruments, New Castle, DE, USA). The sample was heated from 40 to 240 °C at 10 °C/min under a nitrogen atmosphere. The sample was cooled to 40 °C. The sample was again heated to 240 °C at the same heating rate. The glass transition temperature (T_g_) was estimated from the second heating cycle. DSC was performed at the CURF, JBNU.

### 3.13. Pyrolysis GC/MS (Py-GC/MS)

The MWL was derivatized using trimethylsilylation, as described by Pe et al. [43]. Py-GC/MS was then conducted at the CURF under the conditions shown in Table 13.

## 4. Conclusions

The MWL prepared from bamboo grown in Korea was investigated through several structural and thermal characterization techniques, and the results were compared with other bamboo MWLs grown in China. The distinct difference observed in MWL was that it had a higher average molecular weight and a broader molecular weight distribution compared to other bamboo MWLs. Various spectroscopic analyses showed that the MWL was a typical grass lignin but exhibited a very high aliphatic hydroxyl content compared to other bamboo lignins. The MWL also had a considerably lower DTG_max_ (287 °C), which indicated that there were more β-O-4 ether linkages. Through this research on lignin from bamboo grown in Korea, the authors were able to obtain fundamental data on the structural and thermal characteristics of domestic bamboo, and it was confirmed that there were some structural differences from bamboo grown in temperate and subtropical regions.

## Figures and Tables

**Table 1:** Chemical composition of bamboo.

Ash (%)	1.24 ± 0.01
Extracts (%)	
Cold water	5.77 ± 0.02
Hot water	9.85 ± 0.04
1% NaOH	29.69 ± 0.10
Alcohol-benzene	4.77 ± 0.07
Carbohydrate (%)	
Holocellulose	69.25 ± 0.44
α-Cellulose	44.31 ± 0.16
Hemicellulose *	24.94
Lignin (%)	
Klason	27.20 ± 0.09
Acid-soluble	0.51 ± 0.01
Total	27.71

* Hemicellulose (%) = holocellulose—α-cellulose.

**Table 2:** Elemental analyses and methoxyl contents of MWLs.

	Elemental Analysis (%)						Reference
	C	H	O	N	S	OCH_3_	
MWL	58.39	5.66	34.66	0.12	-	20.47	This study
MWL-A	58.78	5.96	34.97	0.28	-	19.48	[19]
MWL-X	63.10	5.67	31.23	-	-	17.74	[20]

MWL: *P. pubescens*, A: *P. acuta*, X: unknown bamboo species. Methoxyl content was calculated by the integrations of the aromatic and methoxyl signals in ^1^H NMR spectrum [18].

**Table 3:** C_9_ formula and formula weight of MWLs.

	C_9_ Formula	Formular Weight (Da)	Reference
MWL	C_9_H_7.76_O_3.23_N_0.02_(OCH_3_)_1.41_	214.17	This study
MWL-A	C_9_H_7.67_O_2.72_(OCH_3_)_1.52_	206.38	[19]
MWL-X	C_9_H_7.53_O_2.65_(OCH_3_)_1.10_	191.59	[20]

MWL: *P. pubescens*, A: *P. acuta*, X: unknown bamboo species.

**Table 4:** Average MW and PDI of Ac-MWLs.

	$\bar{M}$w (Da)	$\bar{M}$n (Da)	$\mathrm{PDI}\;(\bar{M}$$w/\bar{M}$n)	References
Ac-MWL	13,279	4436	3.0	This study
Ac-MWL-A	12,090	5410	2.2	[19]
Ac-MWL-S	6080	3230	1.9	[21]
Ac-MWL-P	6050	3400	1.8	[21]
Ac-MWL-B	7692	4406	1.8	[22]
Ac-MWL-N	9420	7458	1.3	[23]

Ac-MWL: *P. pubescens*, A: *P. acuta*, S, P: stem, pith of *P. pubescens*, B: *Dendrocalamus brandisii*, N: *Neosinocalamus affinis*.

**Table 5:** Assignment of FT-IR spectrum of MWL.

Band (cm^−1^)	Assignments
3441	O–H stretching
2843–2937	C–H stretching in methyl, methylene groups
1718	C=O stretching in unconjugated ketone, carbonyl, and ester groups
1664	C=O stretching in conjugated *p*-substituted aryl ketone
1594	Aromatic skeleton vibration plus C=O stretching; S > G: G_condensed_ > G_etherified_
1503	Aromatic skeleton vibration (G > S)
1462	C–H deformations (asymm in –CH_3_ and –CH_2_–)
1419	Aromatic skeleton vibration combined with C–H in plane deformations
1365	Aliphatic C–H stretching in CH_3_ and phenolic OH
1330	Condensed S and G ring (G ring bound via position 5)
1266	G ring plus C=O stretching (G-methoxyl C–O)
1222	C–O + C–O + C=O stretching (G_condensed_ > G_etherified_)
1160	Typical for HGS lignins; C=O in ester groups (conj.)
1123	Aromatic C–H in-plane deformation (S)
1089	C–O deformation in *sec*-alcohols and aliphatic ethers
1033	Aromatic C–H in-plane deformation (G > S) + C–O deformation in primary alcohols + C–H stretching (unconjugated)
921	C–H out of plane (aromatic ring)
834	C–H out of plane in positions (2 and 6 of S + in all positions of H units)

**Table 6:** ^1^H NMR assignments and distribution of protons per C_9_ structural unit of Ac-MWL.

Ppm	Main Assignments	Ac-MWL
7.20–6.80 *	Aromatic proton in G units	1.05
6.80–6.25	Aromatic proton in S units	0.93
6.25–5.75	H_α_ of β-O-4 and β-1 structures	0.47
5.75–5.24	H_α_ of β-5 structures	0.24
5.20–4.90	H of xylan residues	0.29
4.90–4.30	H_α_ and H_β_ of β-O-4 structures	1.47
4.30–4.00	H_α_ of β-β structures, H of xylan residues	0.88
4.00–3.48	H of methoxyl groups	4.23
2.50–2.22	H of aromatic acetates	0.76
2.22–1.60	H of aliphatic acetates	4.38

* From reference, it was 7.25–6.80, but CDCl_3_ solvent peak was detected at 7.24, thus, the chemical shift was adjusted.

**Table 7:** ^13^C NMR assignment of MWL.

Signal No.	ppm	Assignments
1	170.1	Acetyl C=O in alcohols/phenols
2	169.5	
3	166.3	C-9 in *p*-CE
4	162.0	C-4 in H
5	159.9	C-4 in *p*-CE
6	152.2	C-3/C-5 in etherified S
7	149.2	C-4 in etherified G, C-3 in etherified G with α-CO
8	147.1	C-3 in G, C-3/C-5 in nonetherified S, C-3 in 5-5 biphenyl
9	145.4	C-4 in nonetherified G, C_α_ in *p*-CE
10	138.0	C-4 in etherified S
11	134.9	C-1 in etherified S, C-4 in nonetherified S
12	134.4	C-1 in etherified G
13	133.3	C-1 in nonetherified G
14	132.3	C-1 in nonetherified S
15	130.2	C-2/C-6 in *p*-CE
16	127.9	C-2/C-6 in H
17	125.0	C-1 in *p*-CE
18	119.1	C-6 in G
19	115.8	C-3/C-5 in *p*-CE
20	115.2	C-5 in G, C-3/C-5 in H, C-8 in *p*-CE
21	111.2	C-2 in G
22	106.5	C-2/C-6 in S with α-CO
23	104.2	C-2/C-6 in S, C-4 in β-β resinol
24	103.4	C-2/C-6 in S
25, 26	101.8, 99.5	Residual carbohydrates
27	86.9	C-α in β-5 phenylcoumaran
28	86.1	C-β in β-O-4
29	85.0	C-α in β-β resinol
30	84.5–81.3	C-β in β-O-4
31	75.6	Residual carbohydrates
32	75.3	C-α in β-1, residual carbohydrates
33	73.4	C-α in β-O-4, residual carbohydrates
34	72.2	C-α in β-O-4
35	71.7	C-γ in β-β resinol
36	68.2	NA *
37	64.9	C-γ in β-5 phenylcoumaran
38	62.7	C-γ in β-5 phenylcoumaran, β-O-4 with α-CO
39	60.1	C-γ in β-O-4
40	55.8	OCH_3_ in S and G
41	29.0	CH_2_ in aliphatic side chain
42	20.9	CH_3_ in acetyl

* NA: not assigned.

**Table 8:** Assignments of ^13^C/^1^H correlation signals in the HMQC spectra of MWL.

Notation	*δ*_C_/*δ*_H_	Main Assignments
C_β_	51.5/3.70	C_β_–H_β_ in β-5 phenylcoumaran (E)
B_β_	54.1/3.04	C_β_–H_β_ in β-β resinol (B)
Ome	56.6/3.71	C–H in methoxyls
A_γ_	60.2/3.38–3.89	C_γ_–H_γ_ in β-O-4 (A)
C_γ_	62.8/3.73	C_γ_–H_γ_ in β-5 phenylcoumaran (E)
F_γ_	63.3/4.11	C_γ_–H_γ_ in *p*-hydroxycinnamyl alcohol end-group (F)
B_γ_	71.8/4.16, 72.1/3.80	C_γ_–H_γ_ in β-β resinol (B)
A_α_	72.8/4.85	C_α_–H_α_ in β-O-4 (A)
A_β(G/H)_	82.4/4.34	C_β_–H_β_ in β-O-4 (A) linked to a G/H units
B_α_	85.8/4.64	C_α_–H_α_ in β-β resinol (B)
A_β(S)_	86.6/4.10	C_β_–H_β_ in β-O-4 (A) linked to a S units
S_2,6_	104.8/6.69	C_2,6_–H_2,6_ in syringyl units (S)
S″_2,6_	107.0/7.30	C_2,6_–H_2,6_ in oxidized (C_α_OOH) syringyl units (S″)
S′_2,6_	107.2/7.19	C_2,6_–H_2,6_ in oxidized (C_α_=O) syringyl units (S)
G_2_	111.9/6.97	C_2_–H_2_ in guaiacyl units (G)
H_3,5_	113.8/6.67	C_3,5_–H_3,5_ in H units (H)
G_5_	115.4/6.68	C_5_–H_5_ in guaiacyl units (G)
*p*-CE_3,5_	116.1/6.78	C_3,5_–H_3,5_ in *p*-coumarate (PCE)
*p*-CE_8_	116.2/6.26	C_8_–H_8_ in *p*-coumarate (PCE)
G_6_	119.8/6.80	C_6_–H_6_ in guaiacyl units (G)
H_2,6_	128.3/7.17	C_2,6_–H_2,6_ in H units (H)
*p*-CE_2,6_	130.6/7.48	C_2,6_–H_2,6_ in *p*-coumarate (PCE)
X_2_	73.1/3.05	C_2_–H_2_ in *β*-D-xylopyranoside
X_3_	74.6/3.26	C_3_–H_3_ in *β*-D-xylopyranoside
X_4_	76.0/3.50	C_4_–H_4_ in *β*-D-xylopyranoside
X_22_	74.0/4.49	C_2_–H_2_ in 2-O-acetyl-*β*-D-xylopyranoside
X_33_	75.4/4.78	C_3_–H_3_ in 3-O-acetyl-*β*-D-xylopyranoside

**Table 9:** Hydroxyl and carboxyl contents of MWLs.

	Amount (mmol/g MWL)							References
	Ali OH	Ph OH	C_5_-sub OH + S OH	G OH	H OH	Total OH *	COOH	
MWL	6.74	1.45	0.18	0.51	0.76	8.19	0.23	This study
MWL-N	4.52	1.50	0.28	0.48	0.74	6.02	0.24	[23]
MWL-Y	3.71	1.93	0.59	0.58	0.76	5.64	0.30	[39]

MWL: *P. pubescens*, N: *Neosinocalamus affinis*, Y: unknown bamboo species. Ali: aliphatic, Ph: phenolic, C_5_-sub: C_5_-substituted; * Total OH = aliphatic OH + aromatic OH.

**Table 10:** Composition, onset temperature, and DTG_max_ of MWL (oxidative, inert).

	Condition	Composition (%)				Temperature (°C)		Reference
		120 °C (Volatiles)	400 °C	500 °C	800 °C (Ash/Residue)	Onset	DTG_max_	
MWL	Oxidative	1.9	56.7	34.9	0.3	234	544	This study
MWL	Inert	1.8	89.4	63.9	28.7	235	287	
MWL-N		-	-	-	32.9	-	367	[23]
MWL-Y		-	-	-	21.0	-	360	[39]

MWL: *P. pubescens*, N: *Neosinocalamus affinis*, Y: unknown bamboo species.

**Table 11:** Pyrolysis products and relative composition at 675 °C.

No.	Compound	Type	Formula	RRT ^a^	MW	*m*/*z* ^b^	Relative Composition (%)
1	2-Methylphenol	H	C_7_H_8_O	0.91	108	**108**, 77	3.5
2	4-Methylphenol	H	C_7_H_8_O	0.96	107	**107**, 77	7.0
3	Guaiacol (G)	G	C7H_8_O_2_	1.00	124	124, **109**, 81	10.3
4	4-Ethylphenol	H	C_8_H_10_O	1.17	122	122, **107**	4.5
5	4-Methylguaiacol	G	C_8_H_10_O_2_	1.24	138	**138**, 123, 95	3.4
6	4-Vinylphenol	H/PCA	C_8_H_8_O	1.29	120	**120**, 91	31.7
7	3-Methoxycatechol	S	C_7_H_8_O_3_	1.39	140	**140**, 125, 97	1.4
8	4-Vinylguaiacol	G/FA	C_9_H_10_O_2_	1.50	150	**150**, 135	11.3
9	Syringol (S)	S	C_8_H_10_O_3_	1.57	154	**154**, 139, 93	7.3
10	Vanillin	G	C_8_H_8_O_3_	1.66	152	152, **151**	5.2
11	(E)-Isoeugenol	G	C_10_H_12_O_2_	1.76	164	**164**, 149	2.9
12	4-Propylguaiacol	G	C_10_H_14_O_2_	1.77	166	166, **137**	1.5
13	Acetylguaiacol	G	C_9_H_12_O_2_	1.82	166	166, **151**	2.6
14	4-Allylsyringol	S	C_11_H_14_O_3_	2.02	194	**194**, 91	1.2
15	Syringaldehyde	S	C_9_H_10_O_4_	2.11	182	**182**, 181	4.1
16	Acetosyringone	S	C_10_H_12_O_4_	2.23	196	**196**, 181	2.0

PCA: *p*-coumaric acid, and FA: ferulate; ^a^ RRT: relative retention time, guaiacol as the reference; ^b^ *m*/*z* values in bold: base peak; only *m*/*z* values > 30% of the base peak are included.

**Table 12:** Analysis conditions for GPC.

GPC Configuration	Waters (Acquity APC) System, USA
Columns	Acquity APC 2.5 µm XT 125, Acquity APC 1.7 µm XT 200 (4.6 × 150 mm, Waters, Wexford, Ireland)
Flow rate	0.6 mL/min
Sample injection volume	10 μL
Eluent	THF
Column oven temperature	30 °C
Detector	UV (254 nm: polystyrene standards; 280 nm: sample)
Analysis time	10 min
MW polystyrene standards	Red: 130,000-21,500-6540-1250 Da White: 35,500-9130-2280-266 Da

**Table 13:** Analysis conditions for Py-GC/MS.

Stage	Equipment/Condition	
Pyrolysis	Equipment	Curie-point pyrolyzer (JCI-21, Japan Analytical Industry), pyrofoil (F670, JAI)
GC/MS		GCMS-QP2010 Ultra, Shimadzu
GC	Pyrolysis temp.	670 °C for 5 s
	Interface temp.	300 °C
GC	Column	J & W DB-5MS (30 m × 0.25 mm ID × 0.25 μm, Agilent Techn.)
	Carrier gas	He, 1 mL/min
	Injector/Detector temp.	250 °C
	Split ratio	1:30
	Oven temp.	50 °C (1 min) → ramping (5 °C/min) → 320 °C (5 min)
MS	Ionization	Electron impact method, 70 eV

## Data Availability

The data presented in this study are available in article.
